# Requirements, Limitations and Recommendations for Enabling End-to-End Quality of Context-Awareness in IoT Middleware

**DOI:** 10.3390/s22041632

**Published:** 2022-02-19

**Authors:** Kanaka Sai Jagarlamudi, Arkady Zaslavsky, Seng W. Loke, Alireza Hassani, Alexey Medvedev

**Affiliations:** School of Information Technology, Deakin University, Geelong, VIC 3220, Australia; arkady.zaslavsky@deakin.edu.au (A.Z.); seng.loke@deakin.edu.au (S.W.L.); ali.hassani@deakin.edu.au (A.H.); alexey.medvedev@deakin.edu.au (A.M.)

**Keywords:** context management platforms, quality of context, quality of service, quality of experience, quality-aware selection, IoT ecosystems

## Abstract

Satisfying a context consumer’s quality of context (QoC) requirements is important to context management platforms (CMPs) in order to have credibility. QoC indicates the contextual information’s quality metrics (e.g., accuracy, timeliness, completeness). The outcomes of these metrics depend on the functional and quality characteristics associated with all actors (context consumers (or) context-aware applications, CMPs, and context providers (or) IoT-data providers) in context-aware IoT environments. This survey identifies and studies such characteristics and highlights the limitations in actors’ current functionalities and QoC modelling approaches to obtain adequate QoC and improve context consumers’ quality of experience (QoE). We propose a novel concept system based on our critical analysis; this system addresses the functional limitations in existing QoC modelling approaches. Moreover, we highlight those QoC metrics affected by quality of service (QoS) metrics in CMPs. These recommendations provide CMP developers with a reference system they could incorporate, functionalities and QoS metrics to maintain in order to deliver an adequate QoC.

## 1. Introduction

In the evolving world of pervasive computing, many IoT applications are becoming context-aware by obtaining seamless access to inferred IoT data, which is known as ’context’. Over time, the research community has discussed numerous definitions of context; specifically, as discussed in [1], more than 150 definitions exist. Therefore, to overcome this ambiguity, we consider the widely used definition in [2]: “*Context is any information that can be used to characterise the situation of an entity. An entity is a person, place, or object that is considered relevant to the interaction between a user and an application, including the user and applications themselves*”. The same work defines the context-aware application as “*context-aware applications use context to provide relevant information and/or services to the user, where relevancy depends on the user’s task*”.

The context-aware IoT applications and the data sources are popularly known as the context consumers (or CCs) and context providers (or CPs), respectively. Furthermore, CCs attain seamless context access (access to desired context from the available CPs) through context management platforms (or CMPs). A CMP mediates between the CPs and CCs: it handles storage and processing tasks, from gathering the CC’s requirements to delivering context back to it. For processing each request of a CC, the CMP performs various interlinked processes, which are together known as the context lifecycle [3]. These processes include context acquisition, modelling, reasoning and dissemination. First, in context acquisition, the CMP obtains the relevant raw data (known as low-level context) from the CPs based on the CC’s requirements. Then, context modelling and reasoning transform the low-level context into meaningful information (known as high-level context). Finally, in context dissemination, the CMP delivers high-level context to CCs. The trio: the CMP, its relevant CCs and CPs, form a context-aware IoT environment.

Initially, the CMPs were domain-specific; a few examples include BDCAM [4] and CaCoMAAL [5] for healthcare, and ambience-assisted living domains. However, advancements in the field led to CMPs such as the context-as-a-service [6], FIWARE [7] and COSMOS [8] that offer generic solutions, i.e., they are domain independent, they are applicable across multiple domains and provide diverse and seamless context access.

As compared by Li et al. in [9], the current CMPs exhibit advanced functionalities such as fault tolerance, interoperability, service discovery, storage, security and privacy. Nonetheless, their ability to deliver the context having adequate "quality of context (QoC)" is still in infancy. QoC was defined in [10] as “*any information that describes the quality of information used as context information*”. Various metrics (e.g., timeliness, accuracy, precision and completeness) represent the QoC associated with context, defining its usability to CCs.

The existing work related to QoC in context-aware IoT environments is mainly focused on designing the following frameworks—(i) QoC-aware selection: the frameworks that proactively select the CPs delivering an adequate QoC; and (ii) QoC measurement: the frameworks that compute the QoC adequacy in CPs’ context responses. Implementing these frameworks as a system is functionally sufficient to find appropriate CPs and perform QoC-assured context delivery to CCs. Nevertheless, such a system is still inferior for incorporating with the CMPs. This is because the factors such as the functional compatibility of such a system, requirements of CC, quality definitions of CP, CMP and network performance impact the QoC. Therefore, each actor (CC, CMP and CP) should exhibit certain functional and performance characteristics according to requirements for attaining adequate QoC. We call this end-to-end QoC-awareness.

Our survey aims to identify and study the factors that affect end-to-end QoC awareness (specifically, determining how they affect QoC and at what instance of the context lifecycle). Based on our analysis, we aim to recommend functional adaptations required in a CMP, CCs and CPs and recommend design considerations for quality-aware selection and measurement frameworks. Next section discusses the background and related work, in which we identify the shortcomings of existing work in enabling the end-to-end QoC-awareness and provide our contributions to address them.

The following is this survey’s organisation. Section 2 provides the background and related work and elaborates on our contributions. Section 3 gives an overview of end-to-end QoC-awareness in context-aware IoT environments and discusses the functional adaptions required from each actor to achieve it. Section 4 discuss QoE metrics and their effects on end-to-end QoC awareness. Section 5 discusses the QoS metrics related to process and network dimensions. Section 6 discusses the QoC-aware selection and measurement models; discusses their limitations in attaining end-to-end QoC-awareness at context acquisition and modelling phases. This section also discusses the impact of each QoS metric on the QoC metrics. Section 7 discusses a concept framework designed to address the limitations of QoC-aware selection and QoC measurement approaches. Finally, we conclude this survey in Section 8.

## 2. Background and Related Work

**Figure 1:**
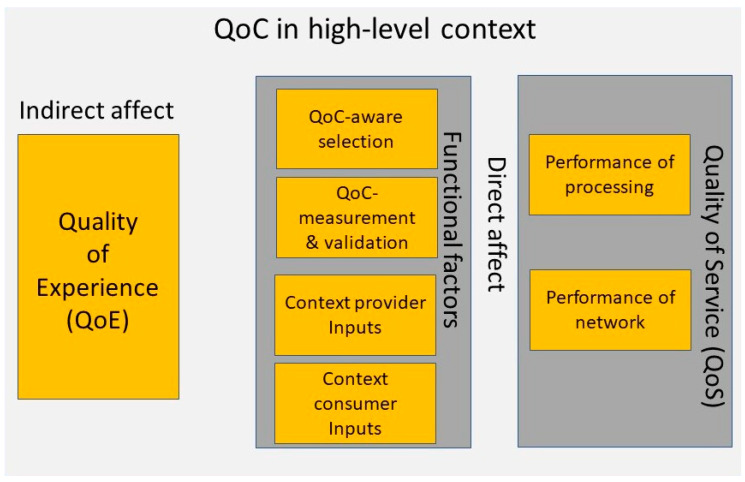
Various factors that affect the QoC in a high-level context. Each block describes a factor. Underlying blocks describe the factors that affect the overarching block’s factor.

There have only been a handful of surveys related to QoC. They either did not discuss or lacked an in-depth discussion on factors that may affect the QoC in context-aware IoT environments. For instance, surveys such as [11,12] have only reflected on QoC metrics’ significance, usability for attaining privacy (in [11]), and performing context reasoning under uncertainty (in [12]). Figure 1 depicts the factors that affect QoC adequacy in context-aware IoT environments.

As seen in Figure 1, the functional and QoS factors directly affect QoC, whereas the factor QoE has an indirect impact. To elaborate on such a classification, the internal factors of a system related to its functionalities or performance affect the context lifecycle processes. Therefore, such factors have a direct effect on QoC. In some cases, the QoC delivered by CMP depends on external factors, which are out of a system’s functional or performance bounds. Such factors have an indirect effect on QoC. A few examples of such factors include the "cost a CC agrees to pay for QoC" and "suitability of context for a particular CC". These factors are mostly related to QoE.

Table 1 depicts the works that have discussed the factors in Figure 1 or considered them in their methods related to QoC. In addition to this, it also reviews their research maturity and current limitations in using them for improving QoC adequacy through end-to-end QoC awareness.

As depicted in Table 1, there is an abundant implementation of QoC-aware selection frameworks. Nevertheless, these frameworks have certain limitations to address in reviewing what is needed for effective implementation in CMPs. For instance, the INCOME framework [14] acquires a similar context from the potential CPs. Then, using QoC Meta-model [18], it measures the QoC in such context and filters out the ones with inadequate QoC.

Nonetheless, the QoC measurement and validation in context responses from numerous CPs, through the filtering process, increase cost and performance overheads. Another example is the framework discussed in [15], which estimates the QoC from CPs based on their hardware characteristics (e.g., sensor accuracy, data push interval) and performs selection. However, the QoC estimates in this approach are performed based on the CPs design time characters, which could be inaccurate considering their susceptibility to quality degradation during runtime. During such situations (upon obtaining inferior QoC during runtime), this framework re-selects the CPs, which results in increased cost and performance overheads. Moreover, these models—and all the models for QoC-aware selection in Table 1—are limited in accurately selecting CPs that satisfy QoE requirements.

The QoC measurement models then compute QoC metrics using metadata in context responses. For instance, using the meta-data—"timestamp at which the CP generated the context"—these models assess the QoC metric "timeliness". The CPs add such metadata while generating the context. The QoC measurement model at CMP computes the QoC metrics upon obtaining the relevant metadata and determines QoC adequacy in context by comparing such metrics with CPs’ QoC guarantees. Nevertheless, the current QoC measurement models discard CPs upon obtaining an inadequate QoC. On the other hand, the CMP may need to compensate the CCs for the incompletion of the context requests that arise due to inadequate QoC. Therefore, a QoC-measurement framework should penalise the CPs concerning their low QoC outcomes. We elaborate on the existing QoC-aware selection and measurement frameworks and their limitations in applicability with CMPs in Section 6 and Section 7.

Using QoC-aware selection and QoC measurement models, a CMP could obtain adequate QoC during context acquisition. Nevertheless, the preservation of such adequacy also depends on the rest of the context lifecycle. For instance, the CMP must generate a high-level context without any delays and missing context attributes during context processing. Moreover, the CMP must also effectively deliver the context without effecting its QoC. Therefore, a CMP must adapt its processing and network resources to exhibit required performance for preserving QoC adequacy. The "quality of service (QoS)" is a dimension that possesses metrics determining network and processing resources’ performance, which affect the QoC during context processing and delivery.

The work in [10] first stated the dependencies between the QoS and QoC. The model in [23] selects the processing resources based on their QoS, along with CPs, to have an adequate QoC during context acquisition and processing. However, this model is limited to processing resources (it does not incorporate the QoS of the network). Moreover, the authors did not discuss the QoC metrics affected by different QoS metrics. Such a reflection is essential for designing QoC-aware resource selection models for resource-constrained platforms.

The works in [10,13] have briefed that QoE increases with QoC adequacy in context delivered to CCs. Nevertheless, the QoE metrics in turn also affect the QoC in context-aware IoT environments. For instance, QoS is a well-known—and the only QoE metric—that directly affects QoC. Hence, it is imperative to identify the QoE metrics and determine their effect on QoC.

Lastly, the works related to QoC measurement have specified the requirements from CCs and CPs to measure and validate each QoC metric. Currently, the CCs and CPs describe their QoC requirements and characteristics, respectively, to the CMPs. The CCs convey their QoC requirements through context queries using languages such as CDQL [22] and PerLa [24]. The CPs describe their context and QoC characteristics using relevant languages (e.g., CSDL [25]). However, for attaining their QoE requirements, the CCs may need to extend the queries with the information relevant to such requirements. Moreover, as the CPs could be third parties, they must specify their costs with the CMP. Such cost declarations assist the CMPs in selecting cost-effective CPs while allowing them to establish a monetary flow with these CPs.

From analysing the above discussion, we gather that delivering adequate QoC to CCs relies on functional and quality characteristics associated with all actors (CMP, CC and CPs). These actors must adapt their characteristics as follows to perform an adequate QoC delivery. A CMP should incorporate QoC-aware selection and QoC measurement framework to attain low-level context with adequate QoC—as part of context acquisition. Moreover, it needs to adopt flexible processing and network resources, which scales its performance to preserve the QoC during context processing and delivery. Furthermore, it also curtails that CPs and CCs define their QoE and cost specifications, respectively. The collective deployment of actors exhibiting such functionalities lead to a system with end-to-end QoC-awareness.

In this survey, we contribute the following for enabling context-aware IoT environments with end-to-end QoC awareness.

- We visualise and provide an overview of a system with end-to-end QoC-awareness through a motivating scenario involving an autonomous car;
- We discuss the significance of each actor and the functional adaptions required of them for enabling end-to-end QoC-awareness;
- We investigate the QoE metrics in CMPs and reflect on their effect on QoC;
- We investigate the QoS metrics related to both processing and network in CMPs;
- We elaborate on existing QoC-aware selection and measurement models and present their drawbacks;
- We present the QoC metrics that may be affected by each QoS metric related to context processing and delivery;
- Lastly, we present a novel concept system that addresses the identified drawbacks of QoC-aware selection and QoC measurement frameworks.

## 3. Significance and Functionalities of Each Actor to Enable End-to-End QoC-Awareness

**Figure 2:**
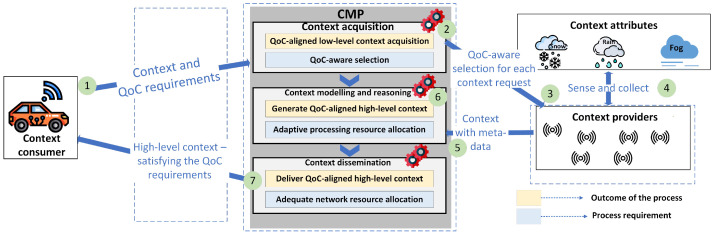
Motivating scenario—the data flow that occurs at each context actor upon enabling the end-to-end QoC-awareness in a context-aware IoT environment. Numbers indicate the order of data flow in such system.

To enable end-to-end QoC-awareness in context-aware IoT environments, all actors need to modify their functionalities and exchange data according to such system’s requirements. In this section, we first visualise and discuss such a system using a motivating scenario involving an autonomous car. We then reflect on the current functionalities of actors and required functional modifications to enable such a system. Figure 2 illustrates the motivating scenario and a system with end-to-end QoC-awareness.

Motivating scenario: The autonomous vehicles equipped with numerous sensors (e.g., LIDARS and image-processing cameras) constantly sense external conditions (e.g., traffic) and adapt their behaviour (e.g., speed). Weather conditions such as rain, fog and snow may affect the sensor inputs [26], thereby influencing the autonomous vehicles’ behaviour. Hence, obtaining the context related to such weather conditions in the area assists them with route selection. For example, by estimating the impact of weather on its sensor inputs, the vehicle decides whether to select that route or choose an alternative with suitable weather.

System with end-to-end QoC-awareness: First, in step 1, the CC (autonomous vehicle) conveys its context, QoC and cost in route-related requirements to the CMP. For instance, the requirements could be following—context attribute: "degree of rainfall", QoC: "timeliness—less than 5 seconds", cost: "3 units". Now, assume that there are multiple CPs providing the required context attribute (degree of rainfall) with varying QoC and costs; such cases are known as the "conflicting situations" [27]. Therefore, in step 2, i.e., at context acquisition, the CMP performs QoC-aware selection, and in step 3, it invokes the CP that satisfies the CC’s requirements.

In step 4, the invoked CP generates and sends the context to CMP. In step 5, the CMP obtains the context with the metadata required to measure the QoC (timeliness in this case) and cost. The CMP then measures the QoC and validates it to align with CC’s QoC requirements. Based on such validity, the CMP uses the context for further steps. In addition to this, it computes the cost of context based on the attained QoC.

Steps 6 and 7 select the required network and processing resources and perform the context processing followed by delivery (dissemination). Here, the CMP selects the resources, as this is cost-feasible (to deliver the context for “3 units” cost), and generates and delivers high-level context by meeting the timeliness and cost requirements.

### 3.1. Expected Functions from a CC

The CCs, in current QoC-aware selection models, specify their entity, context attributes and QoC requirements [14,15] on which the entire system relies. The CCs must thus convey them without any missing or incorrect specifications for the CMP to deliver a correct context and QoC. In addition, the CCs must also extend their requests with QoE requirements (e.g., cost).

### 3.2. Expected Functions from Context Management Platform

According to work in [28], all CMPs related to open environments (those that provide seamless context access from the third-party CPs) contain similar architecture and perform similar functions. We discuss below, the existing processes at each context lifecycle phase in such CMPs, and modifications required for end-to-end QoC awareness.

Context acquisition: This phase begins after the CMP obtains context requirements (e.g., in the form of a context query). The CMP discovers, selects and invokes the relevant CPs based on such requirements. This phase completes after the CMP receives the context responses. Most CMPs select the CPs only based on their ability to complete the context requirements—thus lacking QoC-aware selection. Such limitation can result in the invocation of irrelevant CPs. Moreover, these CMPs are also limited in QoC-measurement procedures, resulting in using the context with poor QoC for processing.

Furthermore, the QoC is also affected by the performance of processes related to context acquisition. For instance, during context requirement gathering (e.g., parsing context queries) from CCs, any missing context attribute or QoC detail due to ineffective processing results in discovering and selecting irrelevant CPs. Therefore, effective QoC-aware selection and measurement frameworks must perform the context accusation. In addition, this phase should also incorporate the adaptive processing resources that produce adequate performance (QoS).

Context modelling and reasoning: These processes are together referred to as “context processing”. Here, the CMP aggregate gathered low-level context depending on their relationships; in the modelling format employed (e.g., OWL, XML, JSON and JSON-LD). The CMP then generates the high-level context in the reasoning phase. An example of high-level context generation based on the above motivating scenario as follows: the CMP may assess the autonomous vehicle-sensors’ resilience to weather conditions (low-level context) at each route. Depending on the result, it may then suggest an ideal route to the autonomous vehicle. The survey in [3] elaborates on the context modelling and reasoning techniques with examples.

The QoS exhibited by the resources performing these processes influences the QoC outcomes. For instance, limited resources may delay the reasoning procedure and affect the context’s timeliness. On the other hand, the functionalities of these processes may still affect QoC. However, such a discussion is beyond the scope of this survey.

The work in [29,30] discussed the adaptive resource allocation to attain QoS in cloud systems. Similarly, the CMPs should adopt the procedures that allocate resources to exhibit the QoS metrics required to preserve the QoC metrics at these phases.

Context dissemination: This is the last phase in the context lifecycle, where the high-level context is delivered to the CC. Using the network channel with the CC as a medium, the CMP delivers context. Therefore, the QoC metrics preserved in the delivered context depends on the QoS of such network. Nevertheless, the network resource selection approaches—to select the network providers that exhibit adequate QoS—for maintaining the QoC level during context delivery is still in infancy. The following example elaborates the effect of the network’s QoS on QoC: an increased “loss rate” (a QoS metric of network) could result in the loss of context attributes, lowering the “completeness” (QoC metric) during the context delivery process. Therefore, for this phase, the CMP should adopt the approaches to identify and select the appropriate network providers that exhibit adequate QoS.

### 3.3. Expected Functions from a CP

For QoC-aware selection: The CPs currently define their context and QoC characteristics in current QoC-aware selection frameworks. These frameworks rely on the match between such definitions and the CCs’ requirements for selection. For instance, in [15], the model relies on the CP/sensor hardware characteristics to perform QoC-aware selection. During registration, the CPs, with their service characteristics, also define the hardware characteristics: accuracy, granularity, time-period, sensor state and sensor range. Using each of these characteristics, the model estimate a particular QoC metric. Hence, by matching such estimates with CCs’ requirements, the framework selects the CPs.

Nevertheless, estimating the QoC metrics from a CP using this approach could be unreliable. This can happen because, firstly, the CPs are prone to performance degradation during run-time; hence, relying on design characteristics such as hardware leads to QoC inadequacies due to run-time inconsistencies. Then, not having the guarantees on the QoC (e.g., SLAs) implies that the CPs are not obligated under any clause to deliver a high QoC; thereby making them untrustworthy for obtaining the same level of QoC from them as estimated.

**Figure 3:**
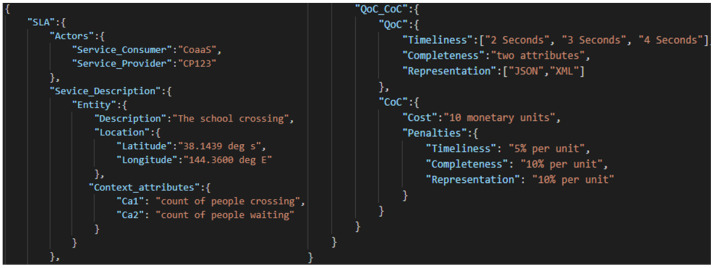
Code snippet of an example SLA template indicating its contents.

Therefore, the CPs should define their QoC guarantees through SLAs, with the penalties for providing the lower than agreed value for each QoC metric. At the same time, they must declare the cost of context on these SLAs for a CMP to make a cost-effective selection. Figure 3 depicts an example SLA, in which the “actors” component represents the CMP and the CP’s details. “Service Description” provides the details of entity and context attributes delivered by the CP. The “QoC and CoC” represent its QoC guarantees, cost and penalty details. Using the latter two components, a CMP can discover the relevant CPs that potentially fulfil the context and QoC requirements.

For QoC measurement: The CPs annotate their context responses by adding the metadata required for QoC computation. The popular QoC measurement models in [15,18,19] rely on such metadata of the context to measure the associated QoC. For instance, the model in [15] computes the timeliness using the metadata: “measurement time of context”. Hence, the CPs must accurately add such metadata (without false or misleading information) for an effective QoC measurement and validation.

This section discussed the functional adaptations that each actor needs to incorporate to attain end-to-end QoC-awareness. Section 4 and Section 5 discuss the QoE and QoS metrics and their effect on QoC adequacy in a system with end-to-end QoC-awareness.

## 4. QoE and Its Effects on End-to-End QoC-Awareness

QoE represents the user satisfaction with the service in service-oriented architectures. Similarly, in context-aware IoT environments, it conveys the satisfaction of CC with CMPs’ services. The QoE indicators related to CMPs are listed in Table 2.

The following works have proposed the resource selection models based on the required QoE. These models are designed for different service-oriented architectures: fog computing [29,30], context-aware computing [32] and IoT [33]. The model in [31] mainly focused on measuring and predicting QoE from a CMP. Nevertheless, despite a few of these works focus on different platforms, the discussed QoE indicators have the same impact on the CMPs.

### 4.1. QoS

All the works–[29,30,31,32,33] have considered the QoS of processing as a factor (metric) influencing QoE. For example, the work in [31] emphasised the importance of QoS for improving QoE. According to the authors, “*Quality of Experience (QoE) is a metric that depends on the underlying QoS along with a person’s preferences towards a particular object or service*”. Moreover, in this work, the authors proposed a model to compute and predict the CC’s QoE using the QoS as a parameter. Generally, the QoS is represented and measured by different metrics (e.g., availability), which we discuss in Section 5.

The following works proposed the resource and process scheduling mechanisms in various domains to attain the required QoS to satisfy the QoE requirements. In [29], the authors investigated the QoS metrics for improving the QoE of fog computing resource users. The deduced QoS metrics include service “availability”, “reliability” and “latency”. Moreover, the authors proposed a model that schedules the fog resources to obtain adequacy in such metrics and improves the QoE. Similarly, the works [30,32] have proposed the scheduling models. Based on the QoE requirements, these models schedule fog and cloud resources for IoT and mobile applications, respectively, to achieve the required QoS.

Furthermore, as discussed in Section 2 and Section 3.2, lower QoS from a CMP in turn reduces the QoC in a high-level context. For instance, consider “scalability”, a QoS metric that reflects the CMP’s performance in dynamically allocating storage and processing resources (e.g., by using dynamic cloud resources such as Amazon EC2 [34]). Consider the task: parsing context queries, in which each query is unique and issued in sequential order. The related component for parsing must scale its processing resources according to the query’s complexity for each query. Insufficient resource allocation would result in missing/incomplete context requests and delays in the parsing procedure. Such incomplete context requests and delays affect context acquisition, causing the CMP to miss the invocation of the required CPs and delays in acquiring the low-level context. Therefore, similarly to the works [29,30,32], and depending on the CC’s QoE requirements, the CMPs must be able to adapt and exhibit the required QoS in order to generate adequate QoC.

### 4.2. Resource Allocation

To attain expected QoC outcomes by having the CMP exhibit an adequate QoS, suitable (or) relevant resources (e.g., CPs and processing resources) must be allocated to each context lifecycle phase. Allocating unsuitable resources affects these phases, resulting in inadequate QoC. For instance, selecting inappropriate CPs for the QoC requirements, in turn, obtains an inadequate QoC during context acquisition. Furthermore, allocating low CPU power for a component that parses the context queries: when this component faces a complex context query, the low CPU power delays context acquisition and affects the succeeding stages. The increased latency affects the QoC metric of timeliness. At the same time, allocating higher than required CPU power to parse the same query, leads to inefficient resource utilisation. Therefore, the CMPs must implement adaptive resource allocation models, QoC and cost-aware CP selection models to efficiently and effectively deliver adequate QoC.

### 4.3. Context Price

The CP’s price to the CMP and the CMP’s price to the CC cause an incurred cost in the CMP and CC. From the IoT data-cost assessment models in [35,36], the data price increases with quality (QoC in our case). Hence, acquiring a high QoC translates to a high monetary cost on the CMP. At the same time, the authors in [37] discussed a pricing model for fog computing platforms, where the price is calculated based on the utilised hardware resources and the cost of IoT data providers. Similarly, the price of a CMP’s service increases based on the cost of resources such as processing resources, network resources and the cost of CPs. Hence, these costs significantly affect the enabling of end-to-end QoC-awareness by increasing the cost when QoS requirements are high while decreasing the cost for low-QoS requirements.

Nevertheless, the high prices result in a decrease in QoE. Hence, the CMP must implement the service selection models for selecting cost-effective CPs and resources. Thus, the QoE is improved by the delivery of a high QoS with the lowest prices. Most works have not considered cost as a QoE metric; except in [31] (as depicted in Table 2), in which the authors used this as one of the QoE metrics in context-aware IoT environments.

### 4.4. Psychological and Cognitive Factors of the End-User

Providing relevant services based on the end-user’s psychological and cognitive factors and their effect on QoE has been introduced in [33]. The following situation illustrates the impact of this metric on the QoC delivered to the CC. Consider a CC, which is a pizza ordering and delivery application. Its users may have different demographic features that drive their budgetary and dietary constraints. They may also have additional situational requirements that drive their service expectations. To attain the QoE of the CC, the CMP must deliver the relevant context that aligns with these requirements and features of their end-users. An example could be, it should provide the context: “only restaurants with vegan options” as the recommendations for users with relevant dietary restrictions. The associated CC retains the information on the psychological and cognitive features based on the user’s profile or by the approaches in [38]. In these approaches, such factors are collected based on "the end-user’s past interactions with the application" or “collecting feedback from the users”.

Nevertheless, it is in the interest of the CC to convey these requirements to the CMP through a context query. Furthermore, during context acquisition, a CMP must select the relevant context that satisfies these requirements. Moreover, it must also validate that acquired context for “correctness”—a metric representing the accuracy of context—to be in accordance with the CC’s requirements before generating high-level context and context delivery. Therefore, the end-user’s psychological and cognitive features significantly impact the end-to-end QoC-awareness.

## 5. The QoS in CMPs and Its Effects on End-to-End QoC-Awareness

As discussed previously, QoS reflects the CMP’s network and processing performance in conducting the context lifecycle. The network’s tasks include obtaining context queries, invocation of CPs, the acquisition of low-level context and high-level context delivery. At the same time, the processing tasks include context query parsing, CP discovery, QoC-aware selection, QoC measurement and validation and high-level context generation. For these tasks, the CMP relies on the network and the processing resources provided by the network and cloud-service providers. Hence, each quality metric related to these network channels and the processing resources reflects the CMP’s QoS and affects the end-to-end QoC. In this section, we discuss the QoS metrics of CMP—related to its processing resources and network service providers—based on which we highlight their effect on different QoC metrics in a system with end-to-end QoC-awareness in Section 6.5.

### 5.1. QoS of Processing

Other than IoT and similar platforms (e.g., CMPs in [21]), the QoS is used as a processing-performance indicator in domains such as cloud computing [39], web services [40] and fog computing [30]. The authors in [41] described the QoS as CMP’s key non-functional characteristic, and proposed a framework for maintaining QoS adequacy in CMPs.

In this sub-section, we review the QoS metrics of IoT platforms; these metrics are also applicable to CMPs—as these platforms share functional similarities. We also identify and elaborate on the QoS metrics specific to CMPs. As listed in Table 3, the significant QoS metrics in CMPs include availability, reliability, scalability, interoperability, security and privacy, service adaption time and QoC.

#### 5.1.1. Availability

Availability represents the accessibility of the platform’s services to the CC at any period, as negotiated on the SLA. The availability is a core QoS metric, the only metric used in famous commercial IoT platforms: Google IoT Core [42]; AWS IoT Core [43]; and Azure IoT [44]. Furthermore, these platforms guarantee the availability as “up-time”, representing service availability during a billing period while agreeing to penalties for its degradation.

According to [21], the dropout rate of the CMP, i.e., the ratio of requests (e.g., context queries) served to the total number of requests represents its availability. In CMPs, the unavailability of both (i) internal resources, i.e., storage and processing resources and (ii) external resources, i.e., CPs, results in service dropouts. Therefore, we deduce that availability is subject to the CMP itself and the CPs it employs.

#### 5.1.2. Reliability

Reliability represents the consistency of a system in terms of its performance. It can be measured as the average time taken by the service to recover from failure, i.e., degraded performance during an availability period [21]. Similarly to availability, reliability is affected by the CMP’s internal processing and the invoked CPs. For instance, a CMP’s context provisioning (e.g., constant updates of a car’s location) to a CC may be disrupted due to the sudden service downtime of the CMP’s processing (cloud) resources or the disruption of context updates from the CP. Reliability metrics at the CMP and CP level are discussed in [15,20], respectively.

#### 5.1.3. Scalability

Scalability represents the platform’s ability to adapt according to the varying process loads of each request and complete them without quality degradation (e.g., QoC degradation). An example defining CMP’s scalability is the ability of the CMP to complete the varying context requests—produced from each context query—by dynamically discovering CPs. According to the survey in [9], most of the existing CMPs such as ACoMs+ [45], FIWARE [7], CA4IoT [28], CoCaMAAL [5], CoaaS [6], BDcam [4], COSMOS [8] are scalable frameworks that complete the context queries by dynamic resource allocation. Nevertheless, upon obtaining QoC-awareness, these CMPs must exhibit the same level of scalability in QoC-aware selection or measurement processes to effectively complete the context queries.

#### 5.1.4. Interoperability

Interoperability represents the CMP’s ability to exchange information between systems (e.g., operating systems). A user-centric context-aware application such as an autonomous car would require context from different CPs. Such context could be the best parking spot, a preferred restaurant or weather conditions for safe travel. Various CPs belonging to different operating systems may produce such context. Hence, despite the CPs’ software characteristics, the CMP must process such context to give seamless context access to the CCs. Moreover, CCs may also have different operating systems that influence their data format requirements. Hence, the CMP must provide the context in a required format to ensure interoperability. In the works on [9], FIWARE [7], CA4IoT [28], CAMPUS [46], SeCoMan [47], CASF [48], CoCaMAAL [5] and BDCaM [4], we find examples of interoperable CMPs.

#### 5.1.5. Security and Privacy

The metrics security and privacy ensure context integrity and privacy, making it accessible to the authorised CCs. The CPs can be of three types [49]: public, private and hybrid. The context from a public CP is published over the CMPs and can be accessed by all CCs. At the same time, the context of private CPs is specific to authorised applications (CCs). Lastly, some context attributes of hybrid CPs are public, whereas some are private. Hence, a CMP must classify the CPs accordingly, and deliver the context to only authorised CCs. The security and privacy level of the CMP is subject to the security framework employed [15] and directly influences the QoC metric: “access right”, which states that context must be delivered only to the applications that the owner of CP specifies.

#### 5.1.6. Service Adaptation Time

The work in [21] discussed service adaptation time as an exclusive QoS metric for context management platforms. Generally, in IoT platforms, the application requirements and the relevant data sources are predefined, forming IoT silos, whereas the CMPs dynamically discover the CPs for the changing needs of CCs. Therefore, the service adaption time represents the time taken by the CMP to adapt its services (to discover the relevant CP and obtain the context) to handle the changed contextual requirements of a CC.

A lower service adaptation time indicates a higher QoS. This metric influences the CMP’s response time, thus indirectly influencing the QoC metric: timeliness.

#### 5.1.7. QoC

The authors in [21] suggested QoC as one of the QoS metrics. Firstly, the QoC is dependent on the platform’s QoS metrics. The QoC is then one of the service expectations of the CCs from a CMP. Hence, based on these aspects, we agree with the authors’ perspective of considering the QoC delivery rate of a CMP as a part of its QoS metrics.

### 5.2. QoS of Network

The QoS of a network indicates the network’s performance during service processing [50]. Its metrics in most service-oriented domains are standard because a network’s functionality is only to establish the communication between the software entities, despite the domain’s type. For instance, the works related to cloud computing [51], Internet of Things [52] and Internet applications [53] has discussed response time, delay, jitter, data rate, bandwidth, loss rate and error rate as the QoS metrics of the network. In context-aware IoT environments, the network connects CMP with CCs and CPs. Nevertheless, we aimed to determine the QoC metrics affected during “context delivery” by different QoS metrics of the network (Section 6.5.2 presents such reflection). Hence, in this section, we only discuss such metrics from the network perspective between the CMP and CCs.

#### 5.2.1. Response Time

The response time is the period between an actor receiving a request and the actor that issued such a request receiving a response. In our perspective, the response time in context-aware IoT environments indicates the duration: starting from CMP receiving a context query, then, processing such query (performing context lifecycle) until the CC receives back the context response from it.

From the above example, along with the “network speed” to carry data between the actors, the response time also depends on the "processing speed" at the CMP after receiving the request. Therefore, we cannot conclude that the “response time” is a QoS metric that entirely relays the network performance; thus, we can rule it out as a QoS metric related to the network. Furthermore, the metric “delay” (discussed in Section 5.2.2) relays the duration of data transfer between the actors. Hence, it can be used instead of response time for assessing the “network speed”.

#### 5.2.2. Delay

The delay represents the time difference between any actor emitting a bit of data and its reception at the receiving end. In context-aware IoT environments, the delay relays the time taken by the network to transfer context from the CMP to CC; typically, the lower delay indicates a higher network performance.

#### 5.2.3. Jitter

The variance in network delay defines the jitter. In context-aware IoT environments, it can be computed as “the variance in delays between the CCs and the CMP”.

### 5.3. Data Rate

The frequency of data exchange between the actors in a session defines the data rate. For example, in CMPs, the frequency at which the CMP sends the high-level context for a query represents its data rate. This metric is particularly applicable to assess the system’s performance in completing those context queries that require a continuous context update (e.g., weather updates) for a particular duration.

#### 5.3.1. Bandwidth

By “bandwidth”, here, we are referring to the maximum allowed data volume to transfer between the actors in each amount of time. For example, in CMPs, the number of context attributes transferred to a CC in a response defines the CMP network’s bandwidth.

#### 5.3.2. Loss and Error Rates

The lost and misrepresented data bits represent the network’s loss and error rates, respectively. Therefore, in CMPs, the lost and misrepresented context attributes in a context response define the loss and error rate in a CMP’s network.

## 6. Methods for Enabling End-to-End QoC-Awareness in CMPs

The existing surveys related to QoC in CMPs (e.g., [11]) is limited to investigating the potential QoC metrics and QoC measurement techniques in the low-level context in CMPs. In contrast, as discussed in Section 3, a CMP must modify its functionalities, from the start to the end of the context lifecycle, for generating and delivering QoC required by the CC.

Therefore, in this section, we first present different QoC metrics that may exist in a context—which a CC may expect—to give an overview of QoC and its usability. Later, we present QoC aware-selection and QoC measurement models—in addition, we also present their limitations in satisfying the QoE metrics during context acquisition. Finally, we reflect on each QoS metric’s affect (of both processing and network) on different QoC metrics during context processing and delivery.

### 6.1. QoC Metrics in CMPs

The QoC metrics indicate the quality properties of the contextual information. Table 4 depicts various QoC metrics discussed in related work.

The terminology differences declared (by “*”) in Table 4 are as follows: the work in [10] addresses confidence as “probability of correctness”, work in [15] defines resolution as “usability”, the work in [54] addresses access right as “integrity”, work in [55] addresses resolution as “range” and work in [57] addresses accuracy as “confidence”.

Although [10] was the first to define QoC metrics, it was limited to five metrics. The works [19,54,55,56,57] did not discuss the entire QoC metrics. The work in [15] has discussed more QoC metrics compared to the rest of the works. However, it does not include the metrics: sensitiveness and trustworthiness; considering them as QoC metrics is arguable. Because, firstly, sensitiveness defines the disclosure level of context, typically assigned by the owners of CPs. This metric shares the similarity with access right, which shares the same definitions (defined below). Secondly, trustworthiness represents the confidence in a CP to provide the context with QoC as guaranteed, thus making it a metric related to the CP’s reputation rather than its context quality.

The metrics in Table 4 apply to both low- and high-level context. The definition, significance and elaboration based on an example of the popular metrics (used in most works) are as follows:

#### 6.1.1. Timeliness

Timeliness represents the delivered context’s degree of validity concerning time.

CCs expect near-real-time information to have an up-to-date context of an entity, using which they can effectively infer situations and make appropriate decisions. Therefore, the CMP must deliver the context that meets imposed time constraints.

For example, if an autonomous car is waiting for context, this may be "the amount of rainfall" at the entity. It requires such context after the rainfall rate reduces under a particular value. In this situation, the CMP must provide the context soon after the rainfall is reduced. The more delays there are in the context generation, processing and delivery after the reduction instance, the more a context’s timeliness decreases.

#### 6.1.2. Completeness

Completeness represents the delivered context’s degree of relevance compared to what is requested.

In context responses, in addition to ensuring the match of context attributes’ quantity with the query’s requirements, it is also essential to ensure the relevance of those attributes with the query. In the context queries, the CCs assign a weight (representing the degree of relevance) to each required context attribute [15] as completeness parameters. Here, the CMP must ensure the highest completeness by delivering the context aligning to the weights specified in each query. The degree of weights’ fulfilment in the context response compared to what was requested defines the completeness adequacy.

For example, an autonomous car (from the motivating scenario in Section 3) along with the context attributes rain, fog and snow, would also specify weights to these attributes in a context query. These weights are specified in descending order based on the car sensor’s vulnerability to the weather elements requested. Hence, a CMP must ensure that the context response contains all the requested weather elements to obtain the highest completeness. However, when there are limited processing resources or cost constraints, the CMP should prioritise the context attributes with higher weights in context delivery.

#### 6.1.3. Confidence

Confidence represents the probability of a match between the delivered context and the ground truth; this metric is also referred to as "probability of correctness" in [58]. As it requires human intervention to accurately determine the ground truth, this metric relies on probable values rather than absolute ones.

CCs entirely rely on context for proactive decision making; hence, it is of utmost importance to ensure that the provided context is correct by measuring the “confidence”.

The following example elaborates on the significance of this metric. A parking lot contains different parking spots (e.g., differently abled, emergency vehicles, regular). Therefore, upon request, a CMP should provide the context: e.g., available parking spots that suits the end-user associated with CC. Thus, by ensuring correctness, the CC’s situational requirements are satisfied.

#### 6.1.4. Significance

Significance represents the value of context for a specific CC [15].

CMP enables seamless context access to all authorised CCs involved in the environment. Nevertheless, the importance of different context attributes varies based on the CC. Hence, significance represents the context value according to the requesting CC.

For example, consider the context: “location of an autonomous car involved in a collision” could have varying significance to different CCs. For instance, it may be more significant to emergency services (e.g., ambulance) than a news channel. Hence, it is essential that the CMP adapts its functionalities (e.g., selecting the CPs of highest quality for CCs with high significance) depending on the significance of context to the CCs.

#### 6.1.5. Resolution

Resolution represents the granularity of contextual information to meet every required detail of an entity by avoiding the noisy context.

The work in [59] classified resolution as temporal and spatial—which determined the applicability of context to a particular time and a particular area, respectively, with precision.

Specific context can be produced with different levels of detail. Hence, the CMP must ensure that the delivered context contains every detail that the CC requires.

For example, if a parking facility issues pricing based on cars’ “duration of parked time” and “type of parking area”(e.g., indoor, outdoor, close to amenities). To effectively issue the price, the facility’s billing system must receive the context with the exact time (including milliseconds) when the car stopped in the parking spot and exact parking area. Therefore, the CMP needs to select CPs and clean the procured low-level context that satisfies the above requirements, ensuring spatial and temporal resolution.

#### 6.1.6. Access Right

Access right represents the extent to which a context item should be accessible to a CC.

Different types of sensor clouds transfer context to the CMP and CCs. Such a context may involve sensitive information that should only be accessed by particular (authorised) CCs and only to a certain extent. Hence, the CMP should have security/integrity preserving mechanisms that ensure context integrity.

For example, if an autonomous cab service “X” obtains its riders’ locations (context) through a CMP. Here, the CMP should ensure that the location is only being shared with the service provider X—hiding the personal details of the rider and thereby maintaining the access rights.

#### 6.1.7. Representation Consistency

Representation consistency represents the degree of context validity according to its format.

CMP would require context from different CPs for responding to a context query. These CPs may produce the context in numerous formats. Hence, the CMP should transform such a context into a format required by the CC for accessibility.

For example, a CP produces and delivers the context in JSON format, while a CC requires it in XML format. Then, a CMP should convert it into XML format while generating a high-level context.

### 6.2. QoC-Aware Selection Models (Requirement at Context Acquisition Phase)

During the context acquisition phase, using QoC-aware selection models/approaches, the CMPs selects the CPs that potentially fulfil the QoC requirements. Based on the current work, we classify these models into three categories: parameter-based models, reputation models and filtering approaches.

#### 6.2.1. Parameter-Based Models

In parameter-based models, the CPs are selected based on their design-time quality characteristics—by estimating their QoC outcomes using these characteristics.

The model proposed in work [15] uses the sensor’s design time characteristics: time-period: “*time interval between two readings of context*”; accuracy: “*extent to which data is correct and free of errors*”; precision: “*degree of exactness with which context is collected*”; granularity: “*degree of detail with which context is collected*”; and sensor range: “*maximum distance for which sensor can collect context*” to estimate the QoC metrics: timeliness, resolution, confidence and resolution. Such design-time parameters are passed to the CMPs during the registration phase of CPs.

Nevertheless, a sensor’s data quality could vary during runtime because of external and internal inconsistencies. For instance, during heavy rainfall and network issues, the QoC in the context may degrade. During such situations, a CMP is required to reselect the CP to fulfil the context request, which would increase the execution time and processing costs, resulting in degraded QoS and an increased processing cost of CMPs.

#### 6.2.2. Reputation Models Based on Social IoT

The works [16,17,60] discussed the selection models based on social IoT or SIoT. The CCs directly select the CPs in these models. Here, every application, i.e., CC, maintains the reputation of CPs from which it received the context. Therefore, if these CCs are looking for a particular context service, they first look for a suitable CP in their repository. Upon not finding it, they rely on their peers’ recommendations. Nevertheless, the existing CMPs abstract the CPs from CCs, making these models unsuitable.

The model in [16] may address the drawback mentioned above. In addition to maintaining the reputation at the CC-level, the CMP also holds the reputation of CPs in this model. When a CC cannot find a suitable CP through its peers’ recommendations, it requests the CMP for a recommendation. Nevertheless, in this model, CMP assesses the reputation in a generalised way, i.e., by collecting the feedback on a CP from various types of CCs. On the other hand, the CC’s satisfaction with the provided context depends on its end-user’s satisfaction, which depends on their psychological and cognitive factors.

The following example elaborates on the effects of selecting CPs based on the reputation assessed in a generalised way on the CCs’ QoE, i.e., because of the impact of its end-users’ "psychological and cognitive factor". When autonomous cabs require to make traffic-related decisions (e.g., route selections), they may require context with adequate accuracy, which depends on their rider’s type. For instance, they may require a higher accuracy while serving a doctor attending an emergency when compared to serving a teenager on a fun ride. While the CCs possess such a diverse group of end-users, the CMP relying on reputation among CCs in unrelated situations makes the selection basis overly generalised. For instance, selecting CPs based on the higher reputation among these cabs with teenagers to serve the cabs with doctors, results in inaccurate-context delivery.

Furthermore, selecting CPs based on their higher reputation among these cabs with doctors for the cabs with teenagers may impose a higher cost on them. Therefore, the QoC-aware selection approaches, especially those based on the opinion models (e.g., reputation, trustworthiness models), should maintain finer-grained scores on the CPs. Moreover, they must select the CPs only based on the reputation among the related CCs (e.g., CCs with similar end-users).

#### 6.2.3. Filtering Approaches

The works [14,20,61] discussed the filtering models/approaches. They overcome the drawbacks of parameter-based models, which is “obtaining inadequate QoC during runtime”. First, the filtering approaches invoke all potential CPs that may satisfy the QoC requirements of a context request. Then, the filtering components deployed by the CP (e.g., the network of smart components [20], the context validator [61]) or those employed by the CMP [14] validate the context responses to filter out the context with inadequate QoC. Nevertheless, despite this approach potentially determining the adequate context, it is redundant in terms of the invocation of suitable CPs, leading to time and cost inefficiencies. For instance, the weather updates of a place can be obtained from the CPs: web APIs and sensors deployed in the area. Here, selecting and invoking a potential API that produces high QoC is more time- and cost-efficient when compared to invoking all CPs and then filtering their context/data to find the one with adequate QoC.

### 6.3. QoC Measurement Models (Consideration at Context Modelling Phase)

QoC measurement models compute QoC in the incoming context streams upon incorporation with a CMP. These models can be used to promote only the context with adequate QoC to the modelling phase. Despite being stand-alone models, we believe they contribute more to the CMPs while implemented with QoC-aware selection models. Due to the unpredictable inconsistencies such as unexpected rainfall, network errors, sensor failures, a CMP cannot wholly rely on the QoC-aware selection approaches to acquire adequate QoC. In such situations, using QoC measurement models, a CMP can measure and validate the QoC in the context responses of those CPs invoked by QoC-aware selection models, thereby always ensuring adequate QoC delivery.

The QoC-measurement models include the OWL-DL ontology [19], QoCIM Meta-Model [18] and other models [15,20]. They follow a similar measurement procedure: the context responses from the CPs are modelled to contain the quality annotations/metadata. Using the relevant annotations, the CMP computes the respective QoC metrics. The QoC-measurement is performed using two elements: QoC indicators and QoC parameters. A QoC indicator is a basis (e.g., formula) for computing its respective QoC metric. At the same time, the QoC parameters are inputs for QoC indicators, which are passed as the context response’s metadata.

#### 6.3.1. The QoC Indicators and Parameters

The works in [15,19,20] explicitly defined the considered QoC indicators and parameters to apply their models. However, despite their similarities in terms of used QoC indicators and parameters, they possess terminological differences. Furthermore, the work [18] only discussed a standard modelling approach to measure and validate all QoC metrics; but does not mention specific metrics to demonstrate the discussed model. The model in [18] is more expressive and usable than others, as subsequently discussed in this section. Table 5 depicts the QoC indicators used in the works mentioned above. Table 6 depicts the QoC parameters used in the works mentioned above as inputs for QoC indicators.

Evidently, from Table 5, all works used similar QoC indicators; the model in [15] used the most indicators of all these works. The authors in [15] topped their model with indicators: “significance” and “representation consistency”. These metrics, respectively, play a significant role in defining the context usability during emergency situations and its processability. Furthermore, the work [15] defined “accuracy” as “reliability”—measured based on the sensor accuracy (hardware feature) and sensor distance from the entity. It indicates the reliability of data, which is similar to the "probability of correctness/confidence" represented in Table 4. Lastly, functionalities of “usability” and “access right” in [15] match with those of sensitiveness and resolution in the model [19]. Hence, the indicators used in models [15,19] are almost similar, except for the significance and representation consistency—whereas the model in [20] used the least number of indicators amongst three works.

In Table 6, each given work has measured the QoC indicator using the parameters given in their joining cell. The parameters related to a few indicators do not apply (N/A) to a few works (e.g., accuracy parameters for [19]) because the models in those works lack these indicators. The parameters are used to compute the given indicators as follows.

#### 6.3.2. Accuracy

The model in [15] referred to the indicator “accuracy” as the “reliability” of context. Nevertheless, it has the same meaning as “accuracy”, which is the correctness of contextual information compared to ground truth. The authors computed this metric as the product of parameters: the ratio of the “distance of the sensor from the entity” and the “maximum distance of sensor from entity until which the sensor’s observation can be trusted” with “sensor accuracy” (hardware characteristic). However, the indicator in this work is limited to a few parameters and did not consider the important parameters that could affect accuracy. For instance, in addition to the distance and sensor accuracy, the external factors such as “adverse weather”, “physical obstacles in the sensing path” and internal factors such as “degraded battery life” could still affect the context accuracy.

Furthermore, the model in [20] computes the accuracy as the ratio of parameters: “collected context value” and the “actual value” (ground truth). For example, consider that according to context: “the amount of occupants in a building is 37” and actual occupants in the building, which is “ground truth is 42”. Thus, the accuracy of context would be 0.8 out of 1. Nevertheless, the indicator in this work relies on the ground truth for the measurement of accuracy, i.e., to determine the parameter’s “actual value”. It requires human intervention to accurately determine such a value. Hence, this indicator possesses limited applicability.

#### 6.3.3. Precision

Precision further quantifies the accurate values in the context for correctness in the presented details compared to what is required. The work in [19] computed the precision of low-level context as the ratio of parameters: “required details from the context" and "total details in the context”. However, [20] computed precision in a high-level context as the ratio of parameters: “collected values” (which are collected context attributes) and “incorrect values” (which are incorrect context attributes in the collected ones). In this survey, as we focused on computing QoC in a low-level context, the indicator discussed in [19] is of greater relevance to us. Hence, we elaborated on it using the example below.

A CC requires the context “timestamp of a rainfall occurrence”. In such a timestamp, along with the units: hours, minutes and seconds, the CC also requires the milliseconds. Hence, “the required details will be 4 units”. Furthermore, the delivered timestamp in the context response only contains the hours, minutes and seconds. Hence, “the provided details will be 3 units”. Therefore, in this case, the ratio of the number of required details to delivered details, i.e., the precision would be “0.75” out of “1”.

#### 6.3.4. Timeliness

Despite terminological differences, all the works [15,19,20] computed this metric using the same parameters and approach. The parameters include “age” [15] (or “currency” [20]) which is “the difference between the timestamp of context collection at entity and the timestamp of context received at CMP”. “validity time” [15] (lifetime [19] or volatility time [20]) is “the validity of context according to time”. Typically, the “validity time” is obtained from CC’s timeliness specifications, or from the sensing intervals of sensors [15] (when the ’time period’ is used instead). The example below elaborates on the timeliness assessment.

A CC requires a car’s location that is collected within 8 seconds after issuing the context query. The CMP determines that the CP must sense and deliver the context to the CMP within “5 s” (which is the “validity time”) for the CMP to complete the context processing and delivery within 8 seconds. In this case, after the CMP receives such context from the CP, if the difference between the “context collection timestamp” at the CP and the “context arrival timestamp” at CMP is greater than 5 s—for instance, “7 s” (which is “age”)—then the timeliness will be 0 (which represents QoC inadequacy). In contrast, if a such difference is within 5 s—for instance “3 s” (which is “age”)—then the timeliness will be 0.6 out of 1.

#### 6.3.5. Completeness

All works measure completeness as the ratio of two parameters: “delivered context attributes” and “required context attributes” in a context response. However, realising that different context attributes may have different importance to the CCs, the model in [15] also takes the weights of context attributes as a requirement specification. Here, the weights specify the importance of context. Therefore, completeness is computed in this work as the ratio of the two parameters: “weights of delivered context attributes” and “total weights”.

Consider the following example to understand the assessment of completeness. An autonomous car requests the context attributes rain, fog and snow. It specified their weights, i.e., “required weights as 0.6, 0.3 and 0.1”. Assume that CMP invokes a CP (e.g., a weather api) which is capable of providing such context. Unfortunately, this CP misses the context attribute “rain” in the context delivery to CMP, making the “delivered weights 0.3 and 0.1”. Therefore, the ratio of both delivered and required weights’ sum, i.e., completeness will be 0.4 out of 1.

#### 6.3.6. Resolution or Usability

Resolution is used in [19]—and called ‘usability’ in [15]—for measuring the spatial granularity level of context. It is measured as a ratio of the parameters “current granularity level” to “required granularity level” of a location.

The following example elaborates on the assessment of resolution. If a CC requests the location of a barber store—requiring the following details: the shop’s co-ordinates, building number, street information and street view image, in which each detail represents an increased level of granularity. Hence, in this case “the required granularity level would be 4”. Furthermore, the CP (e.g., a location api) delivers only the shop’s co-ordinates, street information and building number, resulting in “3 as the current granularity level”. In this situation, the resolution would be 0.75 out of 1.

#### 6.3.7. Sensitiveness or Access Right

The authors in [15,19] used this indicator with the same functionalities but with a different name (sensitiveness in [19], and access right in [15]). This quantifies the context disclosure level to the CCs—as specified by CPs. It is measured as the ratio of parameters: “disclosure level in the existing context” to “disclosure level specified by CP”. The model in [19] uses another metric called access security to determine the level of security in the context communication channel with the CP and estimate the integrity in the context. However, “access-right” could be categorised into a privacy-preserving indicator rather than a QoC indicator. Hence, we do not further elaborate this using an example. The survey in [11] discusses the privacy-preserving techniques and metrics in CMPs.

#### 6.3.8. Representation Consistency

Representation Consistency was used exclusively in the model in [15]. Its outcomes are inversely proportional to the effort to transform the context into a required format. Hence, such a transformation cost of processing resources is used as its computational parameter.

The following example elaborates on this indicator’s assessment. Consider “k” as the processing resources’ cost to transform an incorrectly formatted context attribute; this is represented as “w_ca_”. Therefore, the representation consistency in a context response would be inverse of “number of w_ca_” × k.

#### 6.3.9. Significance

Significance is measured as a ratio of parameters: “delivered critical value”, which is the delivered context’s worth to a particular CC and "required critical value", which is the required context’s worth to a particular CC. Here, the CCs specify the critical values (which are mandatory context attributes required from CMP); hence, the significance is determined by comparing the procured context with those specifications.

The following example elaborates on this indicator’s assessment. Consider an autonomous car (as CP) is involved in a collision. To respond effectively, a paramedic would require the following context attributes: the number of passengers, the location of the car and the fire hazard in the vehicle. Hence, “the required critical value is 3”. If the CP only delivers the context attributes of the number of passengers and the location of the car, then “the delivered critical value becomes 2”. Therefore, in this case, the significance will be 0.75 out of 1.

### 6.4. Limitations of Existing QoC Measurement Models

Firstly, the authors in [15,20] did not address the extensibility/adaptability of their models to add new indicators. However, the models in [18,19] allowed the definitions of new QoC indicators and associated parameters. The CMP developers can customise the QoC indicators and define parameters to compute them using these models.

The QoC indicators discussed in these works aim to measure the context alignment with the CC’s requirements. Such a measurement is inarguably essential for assessing the QoC adequacy to satisfy the CC. However, as the CMPs abstract the CCs and CPs—a CP would potentially negotiate their QoC guarantees and cost with the CMP (e.g., using SLAs as Figure 3). The CMP, in turn, provides the QoC guarantees to CCs. Therefore, in addition to determining the QoC adequacy to satisfy the CC, the QoC measurement models should measure the QoC satisfaction rate of CP compared to its SLA guarantees. Such measurements would assist the CMPs to assess the CP’s reputation and cost (e.g., for QoC-aware selection). Finally, these models limit measuring the absolute values of those QoC metrics that rely on ground truth (e.g., accuracy).

### 6.5. Effects of QoS on QoC (Considerations in Context Processing and Delivery)

#### 6.5.1. Effect of QoS of Processing on QoC Metrics

The authors of [10] have explicitly discussed the effect of QoS on QoC. According to them, the quality of device, i.e., the hardware resource’s quality, directly influences the QoS of an actor (CMP in our case), which in turn affects the QoC outcomes. This implies that an actor should select the processing resources that produces the adequate QoS for acquiring higher QoC. Hence, the QoS of the platform has a significant effect on the QoC.

**Figure 4:**
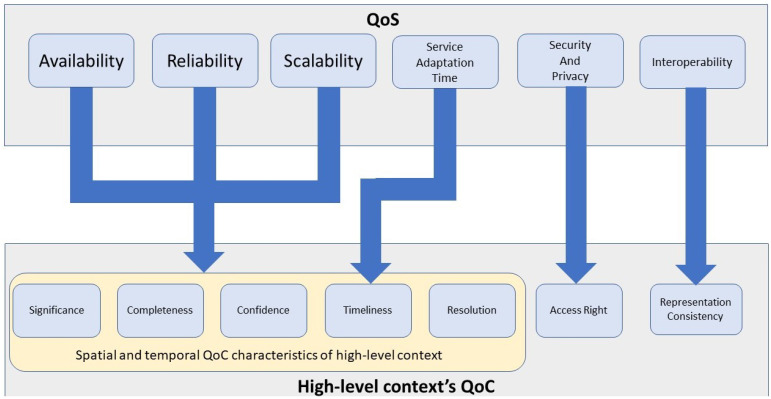
Effect of different QoS metrics (related to processing) on different QoC metrics, where the connecting arrow indicates the effect.

Nevertheless, the literature has substantial shortcomings in identifying the QoC metrics affected by each QoS metric despite such explicit assertions. Finding the affected QoC metrics by different QoS metrics is essential for CMPs, in order to maintain QoC metrics’ adequacy by exhibiting the required amount of QoS. Theoretically, each QoS metric, represented in Table 3, would significantly affect multiple QoC metrics. Hence, based on the definitions of QoS and QoC metrics, we identified those QoC metrics that each QoS metric may affect. We mapped our identifications in Figure 4 and presented our perspective below. Furthermore, the QoS related to the network affects the context delivery phase. The authors in [53] identified those quality metrics that are affected by the network’s QoS. Considering the author’s perspective, we also elaborated on the affected QoC metrics by the QoS of the network during context delivery.

Note: The CPs could also be considered the CMP’s resources with the cloud-processing resources, affecting the QoS the same way as cloud-processing resources. For instance, the unavailability of the required CPs results in the CMP not completing the context query, thereby affecting availability. Furthermore, unreliable CPs may cause service disruptions in the CMPs due to unpredictable disruptions in their low-level context. Hence, for delivering adequate QoS—and in turn, QoC—a CMP should select suitable CPs along with processing resources.

Based on the definition, the QoC metrics of context can be classified into two types: (i) the metrics that are dependent on the context’s spatial and temporal characteristics; and (ii) the metrics that are independent of the same. Firstly, the metrics: timeliness, completeness, resolution, significance and confidence describe the context’s quality based on its temporal and spatial characteristics. For instance, timeliness describes the context’s degree of validity based on time, and thus, is temporal. The other metrics, “completeness”, “correctness”, “resolution” and “significance” are influenced by both spatial and temporal characteristics as follows. Completeness describes the number of required context attributes obtained from an entity. These attributes could describe its (spatial) physical properties (e.g., the number of houses in a particular location) or temporal properties (e.g., the instances of time with specific weather). Furthermore, correctness describes the predicted accuracy of context compared to ground truth; such a context could be related to an event occurring at a particular time (e.g., the volume of rainfall) or a physical entity (e.g., the location of a parked car). Finally, the work in [59] divided resolution as the spatial and temporal, which describes the delivered details related to physical and time properties in a context attribute, respectively, compared to the requested details. Finally, significance describes the importance of context attributes to a specific CC associated with both temporal and spatial characteristics. For instance, a crash reported (context) to paramedics (CCs) contains both the time of the crash and its location.

As depicted in Figure 4, the availability, reliability and scalability affect the QoC metrics related to spatial and temporal characteristics. The seamless context provision of CMPs causes the CCs to expect contextual requirements from different regions at different frequencies. For instance, consider an aeroplane during a flight period. When it is in the tropical areas, it would require frequent updates on rain—over a large area–compared to when it enters other regions. Hence, depending on such situational changes (e.g., when the flight enters a tropical region), a CMP may need to adapt by selecting the CPs and processing resources to address them.

For instance, in the above example, the reduced availability of processing resources when the flight enters a tropical region would result in overheads to handle the increased processing load. This can result in degraded timeliness due to an increased wait time. Then, if the resources are entirely unavailable, the CMP would fail to respond with the required context, resulting in non-completeness and significance degradation. If the CMP lacks the available CPs covering the required geographical area, it will acquire an inferior context, resulting in correctness and resolution degradation. Moreover, the unreliable processing resources and CPs would result in the unforeseen degradation of the same metrics, despite producing high QoC at the start. Finally, a CMP may need to scale its processing resources to increase the context frequency and invoke more CPs for covering more geographical areas. Hence, inferior scalability would increase the processing time and acquire inadequate QoC to satisfy the aforementioned QoC metrics.

Access right and interoperability are the independent metrics of the context’s spatial and temporal characteristics. The CMP’s security and privacy protection mechanisms authorise the CC’s access to the requested context attribute. Hence, the CMP must maintain their adequacy to ensure that only authorised CCs can access context in private clouds—thus preserving the access rights. The interoperability describes the CMPs ability to connect and communicate with CCs and CPs. Hence, the CMP must employ the data transformation mechanisms as part of interoperability standards, to deliver the context in a format required by the CC—thus maintaining representation consistency.

#### 6.5.2. Effect of QoS of Network on QoC Metrics

The survey in [53] explicitly discussed the data quality metrics that are affected by each QoS metric related to networks. These QoS metrics would affect the context during context delivery. We listed the QoC metrics that are affected by each QoS metric related to the network in Table 7.

According to the authors in [53], despite the data type (e.g., text and video), the data quality metrics are influenced by the respective QoS metrics as depicted in Table 7. Specifically, the networks should exhibit an adequate response time, accepted delay and jitter to preserve the timeliness in low-level and high-level context during context transfer between the actors. At the same time, they should exhibit adequate bandwidth accepted loss and error rates to obtain context with reasonable accuracy and precision.

This section provided an overview of QoC, discussed QoC-aware selection models, QoC measurement models, and QoS metrics that may influence various QoC metrics. The first three elements would assist with designing an effective QoC-aware selection and measurement framework based on the presented limitations. The last element would guide the CMP developers to select the processing and network resources for maintaining the QoC adequacy during context processing and delivery. In the following section, we present a concept system that enables effective QoC-aware selection and QoC measurement in CMPs, which can address the limitations of existing frameworks.

## 7. A Concept Framework and Recommendations for Effective QoC-Aware Selection and QoC Measurement

As discussed, most CMPs lack the frameworks and approaches that perform QoC-aware selection or measurement. Such a limitation also applies to popular CMPs that exhibit the most advanced mechanisms. These CMPs include the FIWARE, CA4IoT, BDCAM, CaCoMAAL and CoaaS. FIWARE is an open source project developed by the European Commission. It is widely used in many European projects related to smart cities (e.g., parking) [62] and e-health [63]). Its open source nature offers extensibility to users. CA4IoT and CoaaS are open frameworks (applicable across multiple domains) that provide common platforms for users to query, share and monitor the context. Finally, CaCoMAAL and BDCAM (an extension of CaCoMAAL) are specific to the ambient-assisted living and healthcare domains, respectively. They monitor the individual’s living situation and health to procure the relevant context (e.g., health record) from the Cloud.

Extending the CMPs mentioned above with QoC-aware selection and measurement approaches is advantageous for service selection and delivery. For instance, CMPs could proactively select and invoke quality and cost-effective CPs and cloud-data providers using such an approach. Furthermore, by measuring and validating the context’s QoC and CoC before utilising it, such a framework would preserve the CMPs’ credibility and economy.

First, it is essential to identify the relevant components of the CMP to integrate such approaches. According to work in [28], “*context management middleware solutions always have similar layers with similar names*”, i.e., all CMPs would contain similar components, performing the context lifecycle. Such a typical architecture paves the way to designing generic QoC-aware selection and QoC measurement frameworks. Based on our analysis of CMPs’ functionalities, their components that gather requirements from CCs and accesses CPs are relevant for QoC-aware selection. For sending QoC-assured context responses, CMPs’ low-level context entry points are relevant for QoC-measurement and validation. Table 8 depicts the relevant components for QoC-aware selection (“Gather Requirements” and “Access CPs”) and QoC-measurement (“Receive Context”) in the CMPs given above.

The documentation of FIWARE in [64] and the works related to BDCAM in [4] and CaCoMAAL in [5] abstracted their context manager’s low-level components (Orion Context Broker for FIWARE and Context Manager for BDCAM and CaCoMAAL). Hence, we listed their context managers as a common component for all processes in Table 8. Moreover, BDCAM and CaCoMAAL are domain-specific CMPs deployed as silos (closed systems). The system designers integrate the CPs and CCs beforehand with the CMP, whereas the CMP performs the context lifecycle based on predefined rules. Thus, the requirement gathering components are not applicable (N/A). However, such closed systems could possess CPs producing a similar context with varying quality; thus, they could also benefit from QoC-aware selection and measurement.

According to authors of CoaaS and CA4IoT in [6,28], the components “CSDE” and “PCP” discover and select the CPs based on their QoC and CoC, in the respective CMPs. Nevertheless, the authors did not provide the functional details of such QoC and CoC-aware selection. Moreover, the components “SCP” and “CQA”, receive context with unmeasured QoC or CoC. Thus, the former and latter components should be incorporated QoC-aware selection methods and QoC-measurement models, respectively.

### 7.1. Functional Requirements in QoC-Aware Selection Models

It is important to design the QoC-aware selection models by addressing the drawbacks of existing ones. Section 6.2 highlights such drawbacks; Table 9 summarises them.

The framework “Quality-aware management middleware” uses parameter-based QoC-aware selection model. It performs match-making between the CPs and CCs by estimating the QoC using CPs’ hardware characteristics and comparing it with CCs’ requirements. However, considering that CPs would be prone to QoC degradation during runtime, relying on such design-time characteristics would invoke the CPs that deliver an inadequate QoC.

The “INCOME framework” also match-makes between the CPs and CCs by matching design-time QoC guarantees and requirements; thus, it is unaware of the runtime QoC outcomes. Nevertheless, it obtains the context from all the CPs that satisfies the CC’s context and QoC requirements, and filters their contexts to find the one with an adequate QoC. Hence, it possesses a higher chance of attaining an adequate QoC compared to the parameter-based approach. However, the QoC measurement of multiple CPs causes performance overheads.

Finally, CATSWoTS’s reputation-based selection using SIoT would address the drawbacks of the above approaches by selecting and invoking a CP based on its runtime provisioning ability. In this model, the CC directly assesses and maintains the reputation of CPs. However, such an approach is unsuitable for use in current CMPs, as they prevent communication between CCs and CPs. Furthermore, this model also maintains the reputation at the CMP-level, which is assessed by collecting the QoC values from CCs. The CCs rely on these values when they cannot find the required CP from their peers. However, one of the primary objectives of a CMP is to reduce the processing overheads on CCs. Thus, having the CCs to measure QoC contradicts this motive. Furthermore, the reputation assessed based on the opinions of various un-related CC types would result in the selection of incompatible CPs for the CC-specific requirements.

Nevertheless, reputation-based QoC-aware selection models have fewer limitations (excluding the selection based on peer-level reputation) in terms of efficiency and cost-effectiveness—as these models select CPs based on runtime QoC provisioning. Thus, it is best to use them as a QoC-aware selection approach in a framework by addressing the highlighted limitations. We provide the following recommendations to address such limitations; where (i), (ii) are recommendations related to reputation assessment; (iii) is related to selection based on reputation; and (iv) is related to including cost as a selection parameter.

(i) The framework should compute and maintain the reputation of CPs, indicating their SLA satisfaction rate. Hence, the framework must adopt a QoC-measurement model to compute QoC outcomes in CPs’ context responses. Therefore, by aggregating these outcomes, the framework can compute the reputation of the CPs.

Furthermore, a CP cannot guarantee, and a CMP cannot measure the QoC metrics that rely on a CC’s situational requirements. For instance, "correctness" depends upon the accuracy of context compared to the CC’s requirements.

(ii) The framework should rely on the CC’s feedback to assess the CP’s reputation in satisfying the metrics appropriate to the CC. Here, a CMP must ensure that the CC feedback of a similar type is aggregated as reputation. For instance, the reputation of a CP for a CC with an end-user such as “a doctor attending an emergency” must be separate from the one with an end-user such as “person on a fun ride”.

(iii) The framework should select the CPs by ensuring that they possess a higher reputation among similar CC types, for ensuring satisfaction based on the end-users’ psychological factors and situational requirements (QoE metrics). The framework can achieve such selection by selecting CPs based on the metrics: (i) high reputation in satisfying QoC guarantees (on SLA), where the guarantees match the context request; and (ii) high reputation for the CP with respect to the requesting CC types.

(iv) Neither of the frameworks in Table 9 considers the cost of CPs as a selection factor. There could be multiple CPs with a high reputation for a CC type. Hence, a framework must incorporate a selection approach to select and invoke the CP with a low cost and high penalties (provided for QoC degradation) to achieve cost-effectiveness. We refer to those selection procedures that work based on the cost-related factors of CPs as *CoC-aware selection* approaches.

### 7.2. Requirements in QoC Measurement Models

The existing QoC measurement models are designed to measure CCs’ satisfaction with QoC metrics in delivered context. Nevertheless, reputation and context price rely on the SLA (QoC) guarantees between the CMP and CPs. Therefore, the QoC indicators must be modified to measure the satisfaction of such guarantees. So, the QoC-measurement models can assess the reputation of CPs and context price by mapping the QoC outcomes with the cost and penalties agreed for QoC degradation.

### 7.3. A Concept Framework for Enabling End-to-End QoC-Awareness

**Figure 5:**
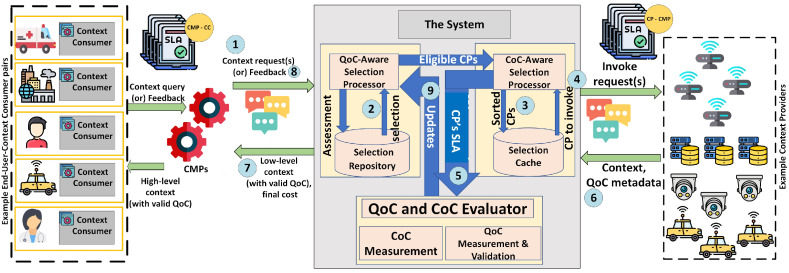
Data flow between the proposed system and the involved actors after deploying it in a context-aware IoT environment.

This sub-section presents our concept system as depicted in Figure 5. It is designed based on recommendations for QoC-aware selection and measurements models in previous sub-sections. The system contains two selection and measurement modules related to QoC and CoC, respectively. The components in the QoC-aware selection module include “QoC-aware selection processor” and “selection repository”. The CoC-aware selection module includes “CoC-aware selection processor” and “selection cache”. The components for QoC and CoC measurement include “QoC measurement and validation” and “CoC measurement”, respectively.

We envisioned deploying the given system as a service (e.g., a standalone application, interacting with different CMPs) instead of directly integrating with a CMP. Such deployment has more benefits. For instance, it extends context availability by matching the context requests from one CMP with the CPs in another, in the case of a lack of required CPs.

The QoC-aware selection processor’s functionalities include QoC-aware selection and reputation assessment. It performs these functions by interacting with the CMP and selection repository. The selection repository contains the SLAs of the CPs and their reputation. The CoC-aware selection processor selects and invokes the cost-effective CP out of eligible CPs (those that satisfy the QoC requirements) from the QoC-aware selection process. In addition to this, it also sends the invoked CP’s SLA details to the QoC and CoC-measurement components. A CP could produce an inferior QoC for a context request, despite possessing a high reputation. To react to such situations, the CoC-aware selection processor uses the Selection Cache, which holds the eligible CPs until successful request completion. The component QoC and CoC evaluator contains the sub-components that are responsible for QoC measurement and validation, and the CoC-measurement, i.e., for measuring the context’s final cost in context responses.

The data flow in the proposed system occurs as follows: after receiving context request(s) in step 1, the QoC-aware selection processor initiates the selection process, i.e., it performs step 2. Depending on the context request’s specifications (containing CC type, context request and QoC requirements), it selects the CP(s) with a high reputation specific to completing them from the selection repository. We refer to such CPs as the “eligible CPs”. Here, the reputation of those eligible CPs is assessed in the reputation assessment process, which is discussed in step 9.

In step 3, the CoC-aware selection processor assesses the cost-effectiveness of each eligible CP based on multi-criteria decision theory (e.g., analytical hierarchy process [65]). The processor assesses the cost-effectiveness by taking the CP’s cost and penalties as the criteria, where having a low cost and high penalties relay a higher cost-effectiveness. After the assessment, the processor sorts and stores the CPs in the selection cache in descending order of cost-effectiveness. In step 4, it subsequently invokes—or suggests that the CMP invokes—the first CP from the sorted list and sends their SLAs to the QoC and CoC evaluator in step 5.

In step 6, the component for QoC measurement and validation receives the context response along with metadata. Then, it measures the outcome of each QoC metric compared to its guarantee on SLA. Furthermore, it validates the QoC by comparing each metric for the match with those defined on the context request and notifies the CoC-aware selection processor regarding the match level. Depending on the notification, the processor clears the selection cache if the outcomes of all QoC metrics match the context request. Otherwise, it invokes the following CP from the cache if any outcomes are inferior to the context request.

The QoC-measurement and validation component then forwards the context for cost measurement, irrespective of context validity. Then, the CoC-measurement component maps the QoC outcomes with the penalties on the CP’s SLA and computes its final cost. It is performed by excluding those penalties applied from the cost given on the SLAs. Then, in step 7, if the received context was valid, it sends the context along with the final cost to generate the high-level context; otherwise, it notifies the CMP of the final cost and discards the context.

In step 8, the QoC and CoC evaluator receives feedback from the CC on metrics relevant to it (e.g., correctness). In step 9, the QoC-aware selection processor receives the CP’s QoC outcomes and CCs feedback from QoC and CoC Evaluator. Finally, the system assesses the CP’s reputation by merging those two values and aggregating them with the existing reputation of the CP. Here, the reputation assessment varies for the following cases.

- *Case 1: QoC metrics violate SLA guarantees, thus, QoC metrics do not match with the context request.* Here, the CP has violated the SLA guarantees by delivering adverse QoC for those metrics defined on its SLA. Hence, the CMP must reduce its general reputation, which is the CP’s reputation in satisfying its SLA guarantees.
- *Case 2: QoC metrics match SLA guarantees and context requests, but the CC is dissatisfied with the delivered context.* Based on the CC’s feedback (e.g., receiving a “dislike” as an indication of dissatisfaction), a CMP should increase the CP’s general reputation while reducing the reputation specific to this CC, so that the CMP can omit this CP from selection in the future for this CC type.
- *Case 3: The QoC metrics match SLA guarantees, satisfy the context request and the delivered context satisfies the CC.* In this case, the CP satisfies both its SLA guarantees and the CC type. Therefore, a CMP should first increase the CP’s general reputation, and additionally, it should increase it further based on the CC’s feedback. Such further increase should be only applied for this particular CC type.

At last, a CMP should also have an SLA with the CC. It should contain the guarantees and implications of high-level context QoC and the cost of context (charged from CC), respectively. However, they are beyond the scope of our discussion.

## 8. Conclusions

The QoC adequacy in a high-level context relies on the functional and quality characters of all actors (i.e., context consumer (CC), CMP and context provider (CP)) in context-aware IoT environments. Existing works and surveys have highlighted the dependencies between these characteristics and their effect on QoC. Nevertheless, it is essential to identify them in a finer-grained manner and adapt the actors’ characteristics to obtain an adequate QoC. We call such adaptation “end-to-end QoC-awareness”. This survey focused on motivating, gathering requirements, reflecting on relevant models and quality dimensions, and discussing the recommendations for enabling end-to-end QoC-awareness in context-aware IoT environments. First, we identified that functionalities of actors, QoC-aware selection and measurement models employed by CMP, quality of experience (QoE) and quality of service (QoS) impact the end-to-end QoC-awareness by affecting the context lifecycle.

Therefore, we discussed each actor’s functional and quality dimensions and dependencies. Then, we reflected on the required adaptations to achieve end-to-end QoC-awareness. We reviewed the QoE metrics in related work and discussed their impact on QoC. Furthermore, we reviewed the QoS metrics related to a CMP’s processing and network performance. Finally, we reviewed the QoC-aware selection and measurement models and highlighted their limitations.

Based on our analysis, we discussed a novel concept system to overcome the drawbacks of QoC-aware selection and measurement. In addition to this, the CMP must exhibit an adequate QoS to generate and deliver high QoC; thus, we have also reflected on the impacted QoC metrics by each QoS metric. These recommendations constitute a perspective that involves CMPs, context-aware application developers and IoT-data providers to achieve end-to-end QoC-awareness. 

## Figures and Tables

**Table 1:** Works discussing the factors represented in Figure 1, research maturity of these factors and limitations.

Factor	Works	Research Maturity	Limitation
QoE	[10,13]	Briefly described	Did not discuss different
		relation between QoE and QoC	QoE metrics’ effect on QoC
QoC-aware selection	[14,15,16,17]	Designed and implemented	Cost and performance inefficiencies;
		frameworks	partially satisfy QoE requirements
QoC-measurement	[15,18,19,20]	Designed and implemented	Lacks quality and cost assurance;
and validation		models and frameworks	lacks cost measurement
Context provider	[15,18,19,20,21]	Described and used input specifications for	Lacks inputs for computing
inputs		QoC-aware selection	the CP costs with respect to QoC
Context consumer	[14,15,19,22]	Described methods and specifications	Did not describe the specifications
inputs		to convey QoC requirements	required to deliver QoC
			as it satisfies the QoE metrics
Performance of	[10,23]	Described and used	Did not specify the QoC metrics
processing		this factor for QoC-aware selection	affected by different QoS metrics
			related to performance
Performance of	[10,23]	Briefly described this factor	Not considered as a factor
network		for attaining adequate QoC	for obtaining adequate QoC;
			did not specify the QoC metrics
			affected by different QoS metrics
			related to network

**Table 2:** The QoE indicators discussed in various works. The tick marks and the blank cells indicate the inclusion and non-inclusion of the respective QoE indicators in the papers.

Reference	QoS	Resource Allocation	Price	Psychological and Cognitive Factors
[31]	✓		✓	✓
[29]	✓	✓		✓
[32]	✓			
[33]	✓			✓
[30]	✓	✓		✓

**Table 3:** Common QoS metrics between IoT platforms and the CMPs and exclusive QoS metrics in the CMPs related to their processing performance.

Common QoS Metrics	Exclusive QoS Metrics
Availability	Service adaptation time
Reliability	QoC
Scalability	
Interoperability	
Security and privacy	

**Table 4:** The QoC metrics discussed in the literature. The first column represents the metrics, and the following ones represent the works. Each marked cell (✓) related to work indicates that the QoC metric in the same row is discussed in that work. The (*) following the check marks in cells indicates that the work defines the QoC metric with a different term.

QoC	Buchholz et al.	Manzoor et al.	Filho et al.	Hossain et al.	Nazário et al.	Neisse et al.	Kim et al.
Metric	in [10]	in [15]	in [19]	in [54]	in [55]	in [56]	in [57]
Precision	✓		✓		✓	✓	
Timeliness	✓	✓	✓	✓		✓	✓
Confidence	✓ *	✓		✓	✓		✓ *
Resolution	✓	✓ *	✓		✓ *	✓	
Completeness		✓	✓		✓	✓	✓
Significance		✓			✓		
Access right		✓	✓	✓ *			✓
Trustworthiness	✓					✓	
Sensitiveness			✓				
Reliability		✓					
Representation		✓					
consistency							

**Table 5:** Comparison of the QoC indicators used in different works, where, the ‘✓’ indicate that the relative QoC indicator is discussed in the respective work. The text in the field indicates term used to address the relative QoC indicator in respective work. The blank fields indicate that the indicator was not used in that work.

QoC Indicator	[19]	[15]	[20]
Accuracy		✓ (Reliability)	
Precision	✓		
Timeliness	✓	✓	✓
Completeness	✓	✓	✓
Resolution	✓	✓ (Usability)	✓
Sensitiveness	✓		
Access right	✓	✓	
Representation consistency		✓	
Usability		✓	
Significance		✓	

**Table 6:** QoC parameters used in different works, where the text in cells indicates the parameter(s) used by the work to compute the indicator in the respective row. The “N/A” shows that the work in the respective column has not considered the indicator in the respective row. "Ca" indicates "context attributes".

QoC Indicator	[19]	[15]	[20]
Accuracy	N/A	sensor accuracy, distance	collected value,
			actual value
Precision	required details, total details	N/A	collected values,
			incorrect values
Timeliness	age, lifetime	age, validity time (or) time period	currency, volatility
Completeness	delivered Ca, required Ca	weights, delivered Ca, required Ca	delivered Ca, required Ca
Resolution	location, granularity level,	N/A	N/A
	required granularity level		
Sensitiveness	disclosure level, accepted disclosure level	N/A	N/A
Access right	current security level, total security level	current disclosure level, accepted disclosure	N/A
Representation consistency	N/A	transformation effort	N/A
Usability	N/A	granularity level, required granularity level	N/A
Significance	N/A	delivered critical values, total critical values	N/A

**Table 7:** QoC metrics affected by different QoS metrics related to the network.

QoS Metric Related to Network	Affected QoC Metric
Response time, delay, jitter	Timeliness
Required bandwidth, loss rate, error rate	Precision, accuracy

**Table 8:** Some functionally advanced CMPs and their components that would communicate with a QoC-aware selection and measurement framework. Each column heading, except ‘CMP’, represents the process of the respective component listed.

CMP	Gather Requirements	Access CPs	Receive Context
FIWARE [64]	Orion Context Broker	Orion Context Broker	Orion Context
			Broker
CA4IoT [28]	Request Manager	Primary Context	Secondary Context
		Processor (PCP)	Processor (SCP)
CoaaS [6]	Context Query Parser (CQP)	Context Service	Context Query
		Discovery Engine	Aggregator (CQA)
		(CSDE)	
BDCAM [4]	N/A	Context Manager	Context Manager
CaCoMAAL [5]	N/A	Context Manager	Context Manager

**Table 9:** Few QoC-aware selection frameworks—along with their selection approaches, description and limitations of these approaches.

Framework	Approach	Description	Limitation
Quality-aware context management middleware [15]	QoC-parameters based selection	Match-making between CCs and CPs based on QoC estimates using CPs’ hardware characteristics	Unawareness of run-time QoC degradation during selection; may require repetition of selection during QoC shortcomings
INCOME Framework [14]	Filtering	Obtains context from all CPs that satisfy requirements; filters them to find the one with QoC adequacy	Unawareness of run-time QoC degradation; QoC measurement for multiple CPs causes performance overheads
CATSWoTS [16]	Reputation based selection in SIoT	Reputation of CPs is maintained at the peer level (at the CC), and at the CMP level; the CCs select CPs based on “reputation” at the peers or the CMP	Advanced CMPs abstract CPs and CCs, hindering the maintenance of peer-level reputation; selection based on general reputation at CMPs may fetch incompatible CPs for CCs; reliance on CCs for QoC-measurement may impose performance overheads

## Data Availability

Not applicable.
